# Can Complex Visual Discrimination Deficits in Amnesia Be Attributed to the Medial Temporal Lobe? An Investigation Into the Effects of Medial Temporal Lobe Damage on Brain Connectivity

**DOI:** 10.1002/hipo.22056

**Published:** 2012-07-31

**Authors:** Sarah R Rudebeck, Nicola Filippini, Andy CH Lee

**Affiliations:** 1 Department of Experimental Psychology, University of OxfordUnited Kingdom; 2 Nuffield Department of Clinical Neurosciences, Functional Magnetic Resonance Imaging of the Brain Centre, University of OxfordUnited Kingdom; 3 Department of Psychiatry, University of OxfordUnited Kingdom; 4 Department of Psychology (Scarborough), University of TorontoCanada; 5 Rotman Research Institute, Baycrest Centre for Geriatric CareToronto, Canada

**Keywords:** resting state networks, diffusion tensor imaging, MRI, hippocampus, perirhinal cortex

## Abstract

It has been suggested that complex visual discrimination deficits in patients with medial temporal lobe (MTL) damage may be explained by damage or dysfunction beyond the MTL. We examined the resting functional networks and white matter connectivity of two amnesic patients who have consistently demonstrated discrimination impairments for complex object and/or spatial stimuli across a number of studies. Although exploratory analyses revealed some significant differences in comparison with neurologically healthy controls (more specifically in the patient with a larger MTL lesion), there were no obvious findings involving posterior occipital or posterior temporal regions, which can account entirely for their discrimination deficits. These findings converge with previous work to support the suggestion that the MTL does not subserve long-term declarative memory exclusively. © 2012 Wiley Periodicals, Inc.

Recent neuropsychological studies have demonstrated complex visual discrimination deficits in human amnesic patients with medial temporal lobe (MTL) damage (e.g., Barense et al.,^2005^; Lee et al.,2005a, b). Together with evidence from nonhuman primate (e.g., Buckley et al.,^2001^; Bussey et al.,^2002^) and rodent (e.g., Bartko et al.,^2007^) lesion research, computational modeling (e.g., Bussey and Saksida,^2002^; Cowell et al.,^2006^), and functional neuroimaging data from neurologically healthy participants (e.g., Lee et al.,^2008^; O'Neil et al.,^2009^; Barense et al.,^2010^), this work suggests that the MTL does not function as a long-term memory system exclusively, but is also critical for processes beyond long-term declarative memory. Although the interpretation of these findings has been debated with regards to long-term declarative memory and working memory processes [for discussion, see Graham et al. (2010) and Lee et al. (2012)], one suggestion is that the perirhinal cortex and hippocampus contribute to higher-order perception and are important for processing complex objects and spatial scenes, respectively (e.g., Murray et al.,^2007^; Graham et al.,^2010^; Saksida and Bussey,^2010^; Lee et al.,^2012^).

Proponents of an exclusive MTL memory system have suggested that complex visual discrimination deficits in amnesic patients may, in fact, be accounted for by damage or dysfunction to regions beyond the MTL that are more commonly associated with perceptual processing (e.g., Suzuki,^2009^). To undermine this explanation, volumetric analyses have demonstrated structurally intact posterior lateral temporal and fusiform cortices in a number of patients that have exhibited complex visual discrimination difficulties (Lee and Rudebeck,^2010^; Barense et al., 2011). Moreover, functional magnetic resonance imaging (fMRI) work has ruled out dysfunction in extrastriate cortical areas that are believed to be important for the perception of scenes, faces, and objects (Lee and Rudebeck,^2010^). Although these findings may argue against the involvement of extra-MTL gray matter damage or dysfunction of individual visual regions, they do not speak to the possibility of disrupted functional connectivity between brain areas or white matter atrophy. To begin to address these concerns, we used fMRI and diffusion tensor imaging (DTI) to examine the integrity of functional resting state networks (RSNs) and white matter connectivity in two of our previous patients, patient HC3 and patient MTL3. Patient HC3 has been previously identified using qualitative visual ratings and volumetric analyses as having selective hippocampal damage (female, age = 51.92 years, education = 10 years), whereas patient MTL3 possesses a larger MTL lesion encompassing the hippocampus and perirhinal cortex (female, age = 65.58 years, education = 10 years; Barense et al.,^2005^; Lee and Rudebeck,^2010^). The two patients demonstrate differing profiles of performance on standard neuropsychological tests of memory [for details, see Lee and Rudebeck (2010)]. On experimental tasks of visual discrimination, patients HC3 and MTL3 exhibit intact discrimination of simple visual stimuli such as color. Patient HC3, however, shows a significant deficit in discriminating complex spatial stimuli but not complex face/object images, whereas patient MTL3 is poor at complex spatial and complex face/object discrimination (Lee et al.,2005a; Barense et al.,^2007^; Lee and Rudebeck,^2010^). We confirmed this pattern of performance for the current study by comparing the performance of patients MTL3 and HC3 on a select number of oddity judgment tasks to a group of 12 age and education-matched neurologically healthy control subjects (female = 7, mean age = 58.58 years, SD = 12.10, mean education = 13.42 years, SD = 2.94; no significant difference in age or education between each patient and the control group as indicated by a two-tailed modified *t*-test for small samples; Crawford and Howell,^1998^; both *P* > 0.1; see Table 1).

**TABLE 1:** Performance (Proportion Correct, Mean with SD Where Relevant) of Patients HC3 and MTL3 and a Control Group on a Set of Oddity Judgment Tasks, in Which the Participants Must Select the Odd-One-Out From an Array of Four Simultaneously Presented Stimuli

	Oddity condition				
	
Participant	Color	Same views faces	Same views scene	Different views faces	Different views scenes
Control group	0.78 (0.08)	0.99 (0.02)	0.97 (0.03)	0.80 (0.11)	0.78 (0.10)
Patient HC3	0.73	1.00	0.93	0.75	0.60*
Patient MTL3	0.72	0.97	0.93	0.55*	0.45*

Stimulus conditions include color [see Barense et al. (2007)], same views faces and scenes, and different views faces and scenes [see Lee et al. (2005a)]. Asterisk indicates significant impairment compared to control group as indicated by a one-tailed modified *t*-test for small samples (Crawford and Howell,^1998^).

For the fMRI and DTI investigations, the patients were compared to 32 neurologically healthy control volunteers that were age and education-matched (female = 17, mean age = 56.67 years, SD = 6.45, mean education = 15.03 years, SD = 4.58; no significant difference in age or education between each patient and controls as indicated by a two-tailed modified *t*-test for small samples; Crawford and Howell,^1998^; both *P* > 0.08). Whole-brain resting state fMRI (rs-fMRI) and DTI data were acquired for each participant on a 3 Tesla MRI scanner and preprocessed and analyzed using tools from the FMRIB Software Library (FSL, http://fsl.fmrib.ox.ac.uk/).

Using independent component analysis (ICA), 8 RSNs were identified in the rs-fMRI data and explored further. These included 7 RSNs described by Beckmann et al. (2005), namely, the medial visual network (MVN), lateral visual network (LVN), sensorimotor network (SMN), default-mode network (DMN), auditory network (AUN), left fronto-parietal network (lFPN), and the right fronto-parietal network (rFPN), and 1 RSN identified by Trachtenberg et al. (2012) termed the posterior hippocampal network (PHN; Fig. 1). In detail, the MVN consists of the primary visual areas along the calcarine sulcus, the bilateral lingual gyrus, and the cuneus. The LVN includes the temporal-occipital fusiform cortex, occipital cortex, and lingual gyrus. The SMN consists of the primary sensory and motor cortices, secondary sensorimotor areas, and the supplementary motor area. The DMN consists of the medial temporal regions, thalamic nuclei, lateral parietal and inferior/middle temporal gyri, cerebellar areas, cingulate, and prefrontal cortex. The AUN involves Broca's area, Wernicke's area, primary auditory cortex of Heschl's gyrus, planum polare, insular and opercular cortex, and the thalamus bilaterally. Finally, the PHN includes the bilateral posterior hippocampus and parahippocampal gyrus, lingual gyrus, amygdala, precuneus, thalamus, brain stem, and temporal pole.

**FIGURE 1:**
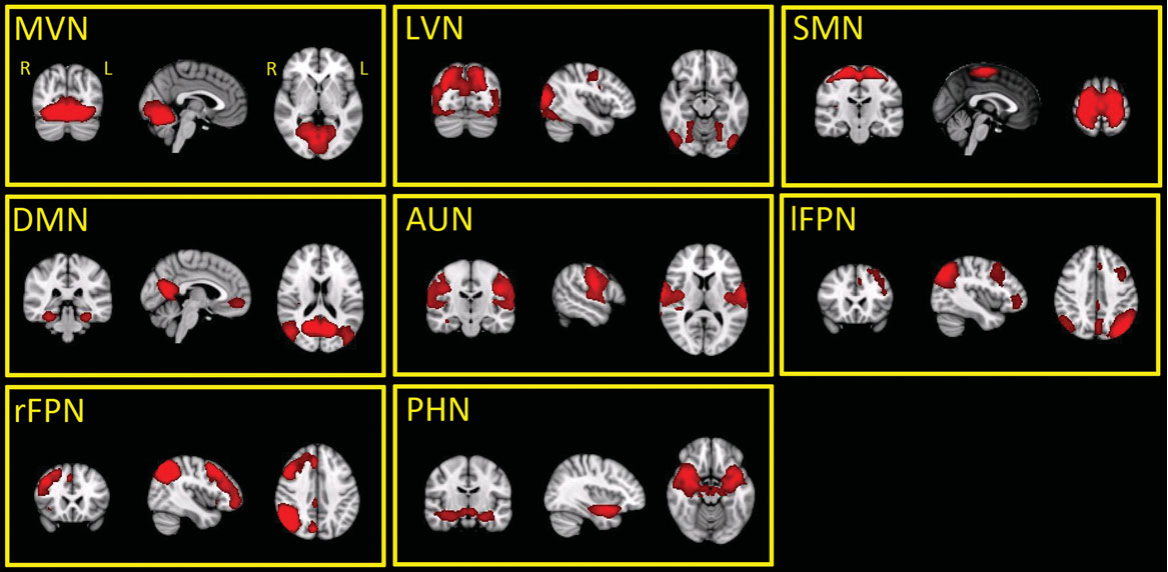
The 8 RSNs identified in the control group (activity rendered on MNI152 template; R, right; L, left). All RSN activity maps were created using a statistical threshold of *z* = 2.3, cluster corrected *P* < 0.05 and further thresholded at different *z*-scores for display purposes. Abbreviations: AUN, auditory network; DMN, default-mode network; lFPN, left fronto-parietal network; LVN, lateral visual network; MVN, medial visual network; PHN, posterior hippocampal network; rFPN, right fronto-parietal network; SMN, sensorimotor network.

In all our imaging analyses, we used a nonparametric permutation-based approach to compare each patient with the control group. Voxel-wise comparisons revealed no significant differences in functional connectivity between patient HC3 and the control group in any of the identified RSNs. In contrast, a decrease in functional connectivity was observed in patient MTL3 compared to controls in relation to the PHN. There was a significantly decreased degree of co-activation between regions that comprise the PHN, in particular the parahippocampal cortex, posterior hippocampus, and thalamus in the right hemisphere, as well as significantly decreased functional connectivity between the PHN itself and regions beyond this RSN, including the right insular cortex, planum temporale, middle temporal gyrus and inferior lateral occipital cortex, and the bilateral anterior and posterior cingulate gyri (see Fig. 2 ). There were no other significant differences between patient MTL3 and the control group in any of the other identified RSNs.

To investigate any differences in gross white matter morphology between each patient and the control group, voxel-based morphometry (VBM; Ashburner and Friston,^2000^) of white matter was carried out on the participants' T1-weighted structural images. This revealed that there was a trend toward a significant decrease in white matter in patient MTL3 throughout the MTL and fornix bilaterally, the right thalamus, and the left and right temporal poles (corrected *P* = 0.059, cluster 1 voxels = 11,038, *x* = 42, *y* = 0, *z* = −46, cluster 2 voxels = 118, *x* = 60, *y* = −18, *z* = −8; Fig. 3). There were no other differences between patient MTL3 and the controls (in particular in posterior occipital and temporal regions), nor were there any significant differences between patient HC3 and the control group.

**FIGURE 2:**
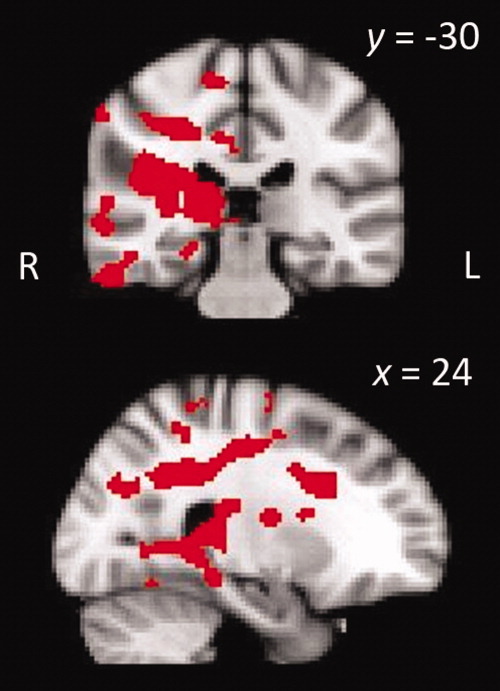
Significantly lower degree of coactivation between regions within the PHN and between the PHN and areas beyond this network in Patient MTL3 (activity rendered on MNI152 template, cluster corrected *P* < 0.05; R, right; L, left).

**FIGURE 3:**
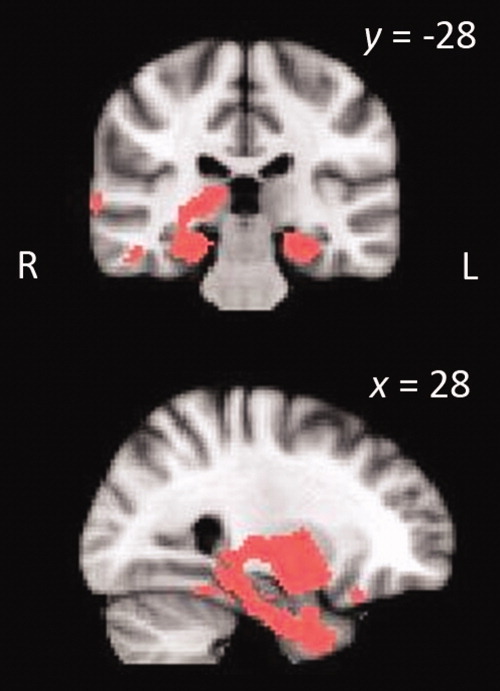
Gross white matter differences between Patient MTL3 and controls as revealed by white-matter VBM (rendered on MNI152 template, cluster corrected *P* < 0.06; R, right; L, left).

To investigate any differences in white matter microstructure integrity between each patient and the controls, we examined fractional anisotropy (FA; Basser et al.,^1994^), as derived from the DTI data, using tract-based spatial statistics (TBSS; http://fsl.fmrib.ox.ac.uk/tbss/; Smith et al.,^2006^). A whole-brain analysis was carried out in the first instance as an exploratory investigation, and this revealed no significant differences in FA between either patient and the control group. A more focused region of interest (ROI) approach was then adopted by focusing on the main white-matter tract between the MTL and medial diencephalon, namely the fornix, and a major white-matter tract connecting the temporal and occipital lobes, namely the left and right inferior longitudinal fasciculi. Neither patient demonstrated any significant differences in FA compared to controls in the left or right inferior longitudinal fasciculi. There was a trend towards a significant decrease in FA in patient HC3's fornix in contrast to controls (corrected *p* = 0.060; cluster 1 voxels = 71, *x* = 0, *y* = −5, *z* = 11; cluster 2 voxels = 21, *x* = 3, *y* = −18, *z* = 15), while patient MTL3 was found to have significantly decreased FA in the fornix body and column (corrected *P* = 0.05; cluster 1 voxels = 62, *x* = 0, *y* = −5, *z* = 11; cluster 2 voxels = 13, *x* = 0, *y* = −2, *z* = 7; cluster 3voxels = 11, *x* = 1, *y* = 1, *z* = 1; Fig. 4).

**FIGURE 4:**
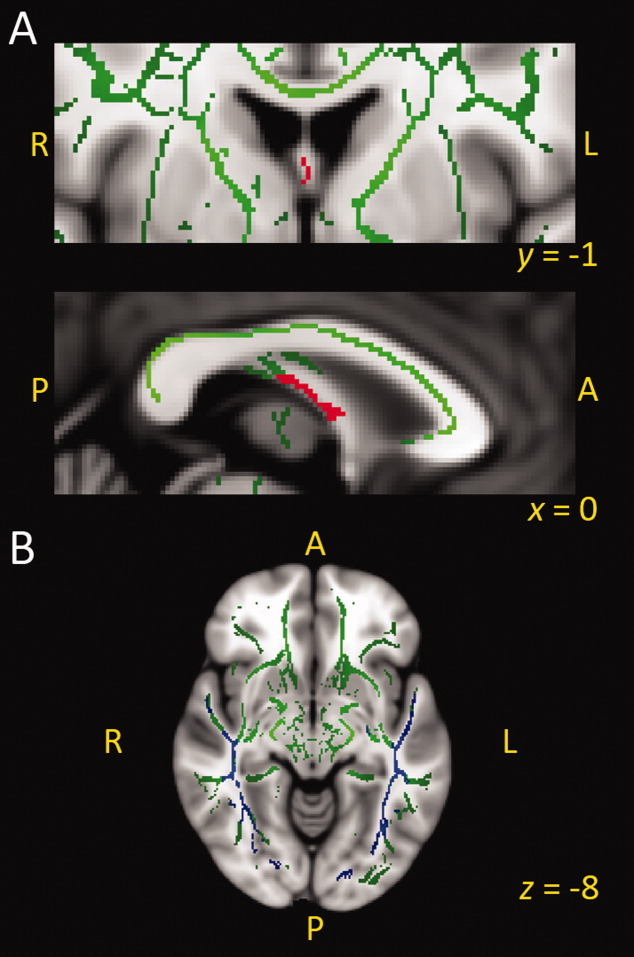
A: Significant difference in FA in the fornix of patient MTL3 (shown in red) in comparison with controls (cluster corrected *P* < 0.05 fornix ROI); (B) No significant differences were observed in the left or right inferior longitudinal fasciculus (shown in blue) in patient MTL3 or patient HC3. Both images overlaid on MNI152 template and white matter skeleton (green). Abbreviations: R, right; L, left; A, anterior; P, posterior.

In summary, the current data reveal no obvious findings to suggest that the complex visual discrimination deficits demonstrated by patients HC3 and MTL3 can be attributed entirely to dysfunction or damage to regions typically associated with visual processing. Neither patient HC3 nor patient MTL3 showed any significant differences compared to controls in functional networks encompassing visual cortical regions (i.e., MVN and LVN) or in white matter in occipital or posterior temporal regions. Although one must express caution in making a conclusion on the basis of a null result, it is the combination of the current data with existing volumetric MRI data, functional MRI of extrastriate visual areas (Lee and Rudebeck,^2010^), and the fact that both patients do not exhibit difficulties on standard neuropsychological tests of perception or demonstrate problems with the discrimination of simple visual stimuli (Lee et al.,2005a; Barense et al.,^2007^) that lends support to the idea that the MTL contributes to higher-order visual perception (Murray et al.,^2007^; Saksida and Bussey,^2010^).

The demonstration of significantly reduced co-activation of a number of brain regions within the PHN and between the PHN and areas beyond this network in patient MTL3 is likely to reflect the relatively large brain lesion in this patient encompassing the MTL structures in both hemispheres (including the amygdala, hippocampus, perirhinal cortex, entorhinal cortex, and parahippocampal cortex), and the temporopolar cortex, anterior fusiform gyrus, and anterior lateral temporal cortex in the right hemisphere [see Lee and Rudebeck (2010)]. Although the precise functional implication of this finding is unclear, it is not implausible to suggest that this decrease in co-activation may, to some degree, contribute to the cognitive deficits that have been observed in patient MTL3. Perhaps surprisingly, no significant differences in RSNs incorporating the hippocampus (e.g., PHN and DMN) were found in patient HC3 compared to controls, despite the presence of a significant hippocampal lesion in this patient. Because of our single-case approach and consequently reduced statistical power, we adjusted our statistical thresholds accordingly when analyzing the patients' data (see Detailed Methods). One cannot, however, rule out the possibility that there are slight changes to functional connectivity following focal brain damage, which can only be detected reliably when larger groups of patients are contrasted to controls. An additional factor that deserves consideration and further investigation is the possibility of brain recovery over time following the occurrence of nonprogressive brain damage [e.g., as seen following stroke; Dijkhuizen et al. (2012)]. Both patients HC3 (1991) and MTL3 (1993) first presented almost 20 years before the current study, and particularly in the case of a smaller focal lesion in patient HC3, there may have been significant changes in functional brain connectivity from the time the lesion occurred to the present day.

To investigate any potential differences in white-matter microstructural integrity, we used a voxel-based approach (i.e. TBSS) first at the whole-brain level, followed by the use of regions of interest (ROI). The former allowed us to carry out an exploratory analysis across the entire brain without delineating individual regions and critically, this revealed no obvious differences in either patients compared to controls in posterior temporal and occipital regions. A more sensitive ROI approach of examining the main white-matter connectivity between the temporal and occipital lobes also yielded no significant differences, with only significantly decreased FA found in patient MTL3 when a fornix ROI was used. It is important to note that although TBSS is sensitive to local changes in white-matter integrity, it does not provide direct insight into subtle changes in long-range connectivity (Smith et al.,^2006^), which requires the implementation of tractography-based connectivity analyses that are significantly more time demanding. Future tractography analyses may provide additional information and detect small changes that our current methods are insensitive to, although by nature of being selective to individual tracts, they have the disadvantage of not being as regionally comprehensive as the TBSS approach adopted here.

To conclude, we have conducted a number of exploratory analyses on the brain connectivity of two patients with MTL damage who have demonstrated significant impairments on complex visual discrimination tasks and found no obvious reasons to suggest that these impairments are due entirely to damage or dysfunction to regions beyond the MTL. Although further work may be necessary to uncover subtle changes in brain connectivity following MTL lesions (i.e., group studies and systematic tractography), it is the convergence of the current data with other findings (i.e., volumetric MRI, fMRI of extrastriate areas; neuropsychological profile) that strongly support the notion that the MTL is critical for higher-order perceptual processing. Moreover, in our extensive use of functional and structural imaging data in the present study and previous work (Lee and Rudebeck,^2010^), we have already gone further to characterize our patients than is common practice in studies of memory processing in amnesic patients with focal MTL damage.

## DETAILED METHODS

This research received ethical approval from the Oxfordshire Research Ethics Committee (07/H0604/115; 08/H0606/133), and all subjects gave informed written consent before taking part. All participants were scanned using a 3 T Siemens Trio scanner with a 12-channel head coil at the University of Oxford Centre for Clinical Magnetic Resonance Research. Whole-brain rs-fMRI data were acquired using gradient echo-planar imaging to acquire T2*-weighted volumes with blood oxygen level-dependent (BOLD) contrast. Each data set consisted of 180 volumes, with 34 axial slices per volume, angled away from the eye balls to prevent image ghosting [voxel size = 3.0 × 3.0 × 3.5 mm, repetition time (TR) = 2,000 ms, echo time (TE) = 28 ms, flip angle = 89°, field of view (FOV) = 192 × 192, acquisition time = 6 min]. During this scan, participants were instructed to lay in dim light with their eyes open and were asked to think of nothing in particular, but reftrain from falling asleep. The DWI dataset consisted of 60 volumes, with diffusion weighting measured isotropically along 60 directions using a *b*-value of 1000 s/mm^2^ (65 axial slices; voxel size = 2.0 × 2.0 × 2.0 mm; TR = 9.3 s; TE = 94 ms; acquisition time = 10 min 30 s). Three volumes without diffusion weighting (*b* = 0 s/mm^2^) were also collected throughout the sequence. A structural T1-weighted image was acquired for each participant using a three-dimensional magnetization-prepared rapid-acquisition gradient echo sequence (TR = 2.04 s; TE = 4.7 ms; flip angle = 8°, voxel size = 1.0 × 1.0 × 1.0 mm, FOV = 224 × 224, matrix size = 174 × 192 × 192).

Rs-fMRI data analyses were conducted using a methodology based on previously published work (Filippini et al.,^2009^). In brief, preprocessing of each rs-fMRI dataset included motion correction, brain extraction, spatial smoothing using a Gaussian kernel of full-width half maximum 6 mm, and temporal filtering with a high-pass filter of 150 s. Because of the extensive lesions in our two patients, particular care was devoted to the registration process. Functional data were aligned to structural images (within-subject) initially using linear registration (FMRIB's Linear Image Registration Tool, FLIRT; http://fsl.fmrib.ox.ac.uk/flirt/) and then optimized using the Boundary-Based Registration approach (Greve and Fischl,^2009^). Structural images were transformed to standard space (Montreal Neurological Institute, MNI, 152 template) using a nonlinear registration tool (FMRIB's Non-linear Image Registration Tool, FNIRT; http://fsl.fmrib.ox.ac.uk/fnirt/), and the resulting warp fields were applied to the functional statistical summary images. Moreover, to ensure that the lesions of the patients did not contribute to registration problems between their rs-fMRI and structural scans, masked normalization was applied. First, a mask was created for each patients' lesion by drawing around the lesion outline on the patients' structural MRI scans. The resulting masks were then applied during the registration of each patient's structural scan to standard space using FNIRT.

After preprocessing, the rs-fMRI data from the 32 controls were concatenated into a single 4D data set and analyzed using probabilistic ICA as implemented in Multivariate Exploratory Linear Optimized Decomposition into Independent Components [MELODIC, http://fsl.fmrib.ox.ac.uk/melodic/; for detail, see Beckmann et al. (2005)]. This defined 52 components representing group-averaged networks of brain regions with temporally correlated BOLD signal. Seven RSNs of interest were then selected on the basis of spatial correlation against a set of previously defined maps (Beckmann et al.,^2005^), with an eighth RSN [a posterior hippocampal network recently described by Trachtenberg et al. (2012)] identified visually. The remaining components reflected BOLD signal drift, white matter, and motion artifacts and were therefore discarded.

The dual-regression method (Filippini et al.,^2009^) was used to compare the resting functional connectivity of each patient with that of controls. First, the full set of ICA spatial maps identified in the control group using ICA was used in a linear fit (spatial regression) against the preprocessed fMRI data set of each individual to create matrices describing temporal dynamics for each component in each participant. These temporal matrices were then used in a second linear model fit (temporal regression) against the preprocessed fMRI data set of each individual. This step produced a statistical parametric map for each component for each subject, describing the extent to which each voxel was involved in each component. The single subject component maps were then collated into 4D files (one for each RSN of interest, with the fourth dimension being subject identification), and any differences between each patient andthe control group were investigated using nonparametric permutation-based testing (5000 permutations; Nichols and Holmes, 2002) as implemented in Randomise (http://fsl.fmrib.ox.ac.uk/randomise). A general linear model (GLM) implemented the following *t*-test contrasts: patient MTL3 > controls, patient MTL3 < controls, patient HC3 > controls, and patient HC3 < controls. These contrasts identified any significant differences in functional connectivity within each RSN as well as any differences in functional connectivity between each RSN and regions in the rest of the brain. Because of our single-case approach, we used a relatively low *Z*-score (1.7) to threshold the resultant voxel-wise maps. Significant clusters were then identified using a family-wise-error corrected cluster threshold of *P* < 0.05.

To carry out the white-matter VBM, the T1-weighted structural images first underwent brain extraction (Smith,^2002^) followed by tissue-type segmentation (Zhang et al.,^2001^). The resulting white-matter partial volume images were then warped to Montreal Neurological Institute 152 space using FNIRT. Registered images were then modulated using the warp field to correct for local expansion or contractions and then smoothed with an isotropic Gaussian kernel (FWHM 2 mm). Randomise was then used to carry out permutation-based nonparametric testing, implementing the same GLM and statistical thresholding as described earlier for the rs-fMRI analysis.

For the DTI analysis, each participant's FA data was first aligned into MNI 152 standard space using FNIRT. A mean FA image was then created and thresholded at 0.2 to produce a mean FA skeleton. Each subject's aligned FA data was then projected onto the FA skeleton by searching perpendicular to the skeleton for maximal FA values (assumed to represent tract centers). TBSS (http://fsl.fmrib.ox.ac.uk/tbss/; Smith et al.,^2006^) was then used in conjunction with Randomise to carry out a voxel-wise statistical comparison between the FA maps of each patient and that of the control participants, using the same GLM and statistical thresholding as that used for the rs-fMRI analysis. An exploratory analysis was carried out by conducting TBSS at the whole-brain level, followed by ROI analyses focused on the fornix, and the left and right inferior longitudinal fasciculi. The left and right inferior longitudinal fasciculus ROIs were identified and created using the John Hopkins University white-matter atlas (http://cmrm.med.jhmi.edu/; mask restricted to a probability threshold of ≥10%). Because this atlas does not delineate the fornix as an individual tract, we used multifiber probabilistic tractography (Behrens et al.,^2003^) to track the fornix in each participant on the basis of previously published methodology (Ringman et al.,^2007^; Voets et al.,^2009^) and created a group mean mask. The voxel within the fornix with the highest FA value was used as the seed for probabilistic tractography, and the resulting tracts were thresholded at 90% to ensure only tracts within the fornix were included. Each participant's fornix tract was then registered linearly to their structural image and then optimized using BBR. The outputs from this step were then transformed to standard space using FNIRT and combined across all participants to create a group fornix mask, which was thresholded at 25%.
